# Research agenda to advance anhedonia assessment, understanding and treatment: an ECNP-GALENOS expert meeting report

**DOI:** 10.1136/bmjment-2026-302843

**Published:** 2026-08-13

**Authors:** B W J H Penninx, Martien J H Kas, Gerard R Dawson, Christoph U Correll, Catherine J Harmer, Derek L Buhl, Michele De Prisco, James Downs, Dennis Hernaus, Seth C Hopkins, Masud Husain, Shaun Johnson, Ruth Mckernan, Edoardo G Ostinelli, Diego A Pizzagalli, Christopher R Pryce, Andreas Reif, Rudy Schreiber, Alessandro Serretti, Spyridon Siafis, Elizabeth Tunbridge, Daniel Umbricht, Christiaan H Vinkers, Andrea Cipriani

**Affiliations:** 1 Department of Psychiatry, Amsterdam Public Health and Amsterdam Neuroscience, Amsterdam UMC, Vrije Universiteit, Amsterdam, The Netherlands; 2 Groningen Institute for Evolutionary Life Sciences, University of Groningen, Groningen, The Netherlands; 3 P1vital Products Limited, Howbery Park, Wallingford, UK; 4 Department of Psychiatry and Molecular Medicine, Donald and Barbara Zucker School of Medicine at Hofstra/Northwell, Hempstead, NY, USA; 5 Department of Child and Adolescent Psychiatry, Charité Universitätsmedizin Berlin, Berlin, Germany; 6 German Center for Mental Health (DZPG), partner site Berlin, Berlin, Germany; 7 German Center for Child and Adolescent Health (DZKJ), partner site Berlin, Berlin, Germany; 8 Einstein Center for Population Diversity (ECPD), Berlin, Germany; 9 Einstein Center for Youth Mental Health (ECYM), Berlin, Germany; 10 Department of Psychiatry, University of Oxford, Oxford, UK; 11 Oxford Health NHS Foundation Trust, Oxford, UK; 12 Neuroscience Precision Medicine, AbbVie, Chicago, Illinois, USA; 13 Hospital Clinic de Barcelona, Barcelona, Spain; 14 Department of Medicine, Faculty of Medicine and Health Sciences, Institute of Neurosciences (UBNeuro), University of Barcelona, Barcelona, Spain; 15 Centro de Investigación Biomédica en Red de Salud Mental (CIBERSAM), Instituto de Salud Carlos III, Barcelona, Spain; 16 Independent Researcher, Cardiff, UK; 17 Department of Psychiatry and Neuropsychology, Maastricht University, Maastricht, The Netherlands; 18 Newleos Therapeutics, Boston, Massachusetts, USA; 19 Nuffield Department Clinical Neuroscience, University of Oxford, Oxford, UK; 20 GAMIAN-Europe, Brussels, Belgium; 21 Operating Partner, SV Health Investors, London, UK; 22 Oxford Precision Psychiatry Lab, NIHR Oxford Health Biomedical Research Centre, University of Oxford, Oxford, UK; 23 Noel Drury, M.D. Institute for Translational Depression Discoveries, University of California Irvine, Irvine, California, USA; 24 Department of Adult Psychiatry and Psychotherapy, University of Zurich, Zürich, Switzerland; 25 Department of Psychiatry, Psychosomatic Medicine and Psychotherapy, University Medical Center Frankfurt, Frankfurt, Germany; 26 Department of Psychopharmacology and Neuropsychology, Maastricht University, Maastricht, The Netherlands; 27 Department of Medicine and Surgery, Kore University of Enna, Enna, Italy; 28 Oasi Research Institute-IRCCS, Troina, Italy; 29 Department of Psychiatry and Psychotherapy, Technical University of Munich, Munchen, Germany; 30 German Center for Mental Health (DZPG), partner site München/Augsburg, Munich, Germany; 31 Boehringer Ingelheim Pharma GmbH and Co KG, Ingelheim, Germany; 32 xperimed LLC, Basel, Switzerland; 33 Department of Psychiatry and Anatomy and Neurosciences, Vrije Universiteit Amsterdam, Amsterdam, The Netherlands; 34 Amsterdam Neuroscience, Mood, Anxiety, Psychosis, Sleep & Stress Program, GGZ inGeest Mental Health Care, Amsterdam, The Netherlands; 35 NIHR Oxford Health Clinical Research Facility, Oxford Health NHS Foundation Trust, Warneford Hospital, Oxford, UK

**Keywords:** Depressive Disorder, Psychopharmacology

## Abstract

Anhedonia, broadly defined as a reduced ability to experience interest or pleasure, represents an important transdiagnostic neuropsychiatric symptom dimension which may benefit from targeted diagnostics and treatments. Different lines of research have proposed that it comprises multiple facets, including deficits in anticipatory (‘wanting’) and consummatory (‘liking’) reward processing as well as reward learning and affects different aspects of life (eg, social, physical, cognitive). Certain facets—more specifically anticipation, motivation and reward learning—likely involve blunted phasic dopaminergic signalling. However, recent meta-analytical evidence of human depression studies indicates that prodopaminergic antidepressants produce relatively small improvements in anhedonia symptoms and suggest that mechanisms beyond dopamine likely contribute to anhedonia. This stimulated an expert meeting to review the literature and define priorities for future research in anhedonia. A central key priority is developing a translational biologically-informed nomenclature and consensus that solves the current mismatch between constructs, paradigms and measures, and mechanisms, which separates discrete reward-related processes such as effort allocation, reward learning and anticipatory interest versus consummatory pleasure. *Clinical research* priorities are improved multimodal measurement tools, integrating neurobiological frameworks (eg, neuroimaging, electrophysiology and liquid biomarkers capturing dopaminergic, glutamatergic, opioid and immunometabolic pathways) and transdiagnostic studies across neuropsychiatric disorders and developmental stages. Innovative trial designs that explicitly target anhedonic phenotypes as a primary outcome and test mechanism-based interventions are also needed. *Translational research* recommendations include back-translation strategies that begin with patient-relevant phenotypes followed by the development of comparable human and animal tasks that target reward-related processes, such as effort allocation, reward learning and anticipatory interest versus consummatory pleasure, improve cross-species behavioural paradigms and enhance methodological rigour and reproducibility. Collectively, these recommendations will help refine the conceptualisation of anhedonia and advance its role within precision psychiatry as a mechanistically grounded target across multiple disorders.

## Introduction

 Mental health disorders display a heterogeneity of symptom dimensions and neurobiological mechanisms that overlap across disorders. At its core, precision psychiatry aims to pinpoint the underlying neurobiological mechanisms responsible for the emergence and persistence of symptoms of mental health conditions.^1^ This approach strives to create diagnostic tools and therapies targeting these mechanisms by leveraging the existing evidence base to provide more targeted symptom management and possibly alter the disease trajectory. The expected goal of precision psychiatry is a more refined and biology-informed diagnostic approach for brain disorders, involving transformational changes and opportunities for drug development, regulators, clinicians and ultimately patients. A related and critically important goal is to eventually guide treatment selection.

Anhedonia has received considerable research attention and is recognised as a top priority by non-profit initiatives, such as the James Lind Alliance (https://www.jla.nihr.ac.uk/). It was originally defined as the reduced ability to experience joy or pleasure from activities that were previously experienced as enjoyable. More recent work has proposed that anhedonia has many facets. Broader definitions also incorporate reduced levels of the inter-related states of interest, anticipation and motivation,^2 3^ with a distinction between *anticipatory* (ie, ‘reduced wanting’) and *consummatory* (ie, ‘reduced liking’) *anhedonia*. Additionally, it seems important to differentiate different domains of life it may affect: for example, *social anhedonia* (ie, not enjoying social interactions or feeling disconnected from others) from *physical or sensory anhedonia* (ie, lack of looking forward to and/or finding pleasure in food, touch, music or other sensory experiences). A large body of evidence in humans and experimental animal models suggests that dysregulated mesolimbic reward pathway, likely involving reduced dopamine signalling, blunted ventral striatum activity and impaired reward interest and learning, all play a key role in anticipatory anhedonia.^4–6^ In people with major depressive disorder (MDD), anhedonia has consistently been associated with poorer psychosocial functioning, reduced quality of life, impaired functioning^7^ and less favourable clinical course and poorer treatment response.^8^ Similarly, in a transdiagnostic mood disorder sample, anhedonia emerged as a robust correlate of quality of life cross-sectionally and longitudinally. Critically, potentiated neural reward responsiveness at baseline predicted greater improvements in quality of life over time, and this improvement was mediated by longitudinal improvements in anhedonia severity.^9^ Moreover, conventional antidepressant treatments often improve mood without fully resolving anhedonia. These findings suggest that interventions specifically targeting reward processing and anhedonia may yield clinically meaningful benefits, supporting the need for studies that evaluate anhedonia as a primary treatment outcome. Finally, while most of this research has been undertaken in the context of MDD, extensive evidence suggests that anhedonia represents a core and transdiagnostic symptom that is present across a wide range of psychiatric and neurological disorders.^10^

The Wellcome-funded GALENOS project (*Global Alliance for Living Evidence on aNxiety, depressiOn and pSychosis: https://www.galenos.org.uk/*) is an international initiative that aims to provide a dynamic and collaborative platform for coproduced living systematic reviews (LSRs) to accelerate progress in mental health research by keeping evidence-synthesis on anxiety, depression and psychosis updated and freely accessible to everyone who needs it.^11^ Using triangulation—a methodology which identifies, evaluates and synthesises a wider variety of evidence, including expertise from people with lived experience and using prompts from large language models—GALENOS LSRs enable a more comprehensive approach to prioritising future research directions and, ultimately, driving improvements in clinical practice.^12–14^

A GALENOS LSR evaluated the effects of pro-dopaminergic drugs on anhedonia in MDD including both human (ie, specific anhedonia items from larger depression assessments, such as the ‘inability to feel’ item from the Montgomery-Åsberg Depression Rating Scale, MADRS) and animal data (eg, sucrose preference tests).^15^ Prodopaminergic drugs (amitifadine, bupropion) were associated with a small reduction of anhedonia symptoms (6 randomised controlled trials, n=2076; Standardised Mean Difference [SMD] −0.24, 95% CI −0.46 to −0.03) and increased sucrose preference in animal models (27 studies; SMD 1.34, 95% CI 0.88 to 1.79). No data were found on reward/reinforcement tasks in humans, and evidence was rated as low to moderate. In the same LSR, somewhat unexpectedly, a network meta-analysis of human studies with patient-level data showed that antidepressants with a non-dopaminergic mechanism of action were associated with a bigger reduction in the MADRS ‘inability to feel’ item than prodopaminergic drugs, and the ‘anti-anhedonia’ effect was potentially independent of the improvement in mood symptoms.^16^ Based on these findings, the precise neurobiological mechanisms of anhedonia in MDD remain poorly understood and likely involve mechanisms and systems that include, but are not limited to, dopaminergic pathways. This empirical observation formed the rationale for a meeting involving multidisciplinary experts in animal and human studies, to review the literature and discuss future research needs in the field of anhedonia.

## Methods

In January 2026, GALENOS and the European College of Neuropsychopharmacology (ECNP) organised a 2-day meeting on anhedonia with 24 multidisciplinary stakeholders (researchers, academics and industry partners with preclinical or clinical expertise and people with lived experience). The meeting was funded by Wellcome and the international participants were selected through an open call. After presenting the results from the GALENOS LSR on anhedonia,^16^ experts were divided in smaller working groups to actively discuss and define pivotal and topical research questions to progress the field of anhedonia. Working groups made recommendations for the next stages of research and how to address these in various fields: clinical, translational and preclinical studies. Wellcome and ECNP staff members participated as observers. Below we report on the main outputs of the meeting, focusing on actionable research steps to advance conceptual and mechanistic understanding of anhedonia and make tangible progress towards precision medicine (table 1).

**Table 1:** Actions needed to advance anhedonia research and treatment

Short-term (<2 years)	
1	Qualitative and Delphi studies to refine concept of anhedonia and its subdomains
2	Develop new subdomain-specific tasks and validated questionnaires where needed
3	Map and harmonise existing (randomised and observational) datasets that include measures of anhedonia
4	Expand GALENOS living systematic review to pharmacological and non-pharmacological treatments for anhedonia
Mid-term (2–5 years)	
5	Validate subdomain-specific anhedonia assessments in human and animal studies using back-translation
6	Use existing datasets for cross-study analyses of associations between anhedonia and cross-disorder clinical/neurobiological parameters
7	Enrich intervention studies using diverse pharmacological and psychosocial treatments with validated anhedonia subdomain measures
8	Conduct new multimodal studies to examine neurobiological mechanisms of anhedonia subdomains
Long-term (>5 years)	
9	Experimental medicine studies and neurobiology-based targeted RCTs in collaboration between academia and industry

GALENOS, Global Alliance for Living Evidence on aNxiety, depressiOn and pSychosis; RCT, Randomised Controlled Trial.

## Presentation

### Clinical research

#### Conceptual refinement of anhedonia

In clinical studies, anhedonia has originally been defined as the lack of experiencing pleasure (anticipatory anhedonia), but it nowadays is also often defined as a state of reduced motivation and interest (consummatory anhedonia). Thereby it overlaps with the concept of apathy, whose core feature is lack of motivation. There is considerable overlap in people with clinically relevant anhedonia and apathy, but there are also individuals who suffer from only one of these. Factor analysis shows that apathy and anhedonia appear to be at least partly separable entities.^17^ Most commonly-used self-report measurement scales of anhedonia (such as the Dimensional Anhedonia Rating Scale (DARS) or Temporal Experience of Pleasure Scale (TEPS)) capture aspects of both anticipatory and consummatory processes. Consequently, confusion arises about the core concept of anhedonia, as it remains unclear what the overlap and distinction between anticipatory and consummatory hedonic components are. In addition, anhedonia can be linked to different aspects of life (affective, cognitive, physical and social) and not all people experience anhedonia in all these aspects.^6^

As anhedonia is clearly not a single, unitary construct, it is unlikely to be driven by a single mechanism. Instead, disruption in multiple biological systems may converge on similar subjective experiences and functional impairments. Therefore, research should be moving away from the generic label ‘anhedonia’ towards a more precise definition based on discrete cognitive and motivational mechanisms, for example, potentially based on the Research Domain Criteria: Reward Responsiveness (eg, reward anticipation, reward satiation), Reward Learning (eg, reinforcement learning) and Reward Valuation (eg, effort allocation).

The inclusion of qualitative studies can help facilitate a more in-depth understanding of subjective experiences of anhedonia, including its functional implications and the treatment preferences of people with lived experience. Similarly, a Delphi study, including people with lived experience, preclinical/clinical researchers and clinicians, could help reach consensus about the conceptual definition and nomenclature of anhedonia, its subdomains and the relevant related dimensions (including fatigue, sedation, attentional impairment, social withdrawal or alexithymia) and whether current questionnaires and rating scales sufficiently capture them. Care must be taken to ensure meaningful lived experience representation, transparent consensus criteria and minimal attrition across rounds, so that any agreement reached reflects genuine conceptual convergence.^18^ Together, these efforts could help inform the development of more accurate and translatable models and paradigms. Importantly, consensus on conceptual definition and nomenclature should be based on a biological framework.

#### Measurement development, validation and harmonisation

Measurement fragmentation has significantly slowed progress. Existing self-report scales capture important but incomplete aspects of the construct.^19^ Some emphasise consummatory pleasure (eg, Snaith-Hamilton Pleasure Scale), others distinguish anticipatory from consummatory processes (eg, DARS or TEPS) and still others differentiate social from physical domains (eg, Chapman Anhedonia Scales). However, few integrate all these dimensions comprehensively. Beyond self-report, assessment should incorporate behavioural paradigms to capture anticipatory and consummatory hedonia as well as other reward-related subdomains. An often-used behavioural effort–reward task is the Effort-Expenditure for Rewards Task, which assesses willingness to expend effort, sensitivity to reward magnitude and probability and cost-benefit decision-making.^20^ This task primarily taps into motivational/anticipatory interest, but not consummatory pleasure. There remains limited information on the correlation of various self-report and behavioural measures of anhedonia. Identification of available cohort and trial datasets with different anhedonia subdomains assessments should stimulate structured, harmonised and cost-efficient cross-cohort analyses that provide insights in intercorrelations of subdomains and associations with neuropsychiatric disease outcomes, comorbidity and treatment effects.

At the same time, the field would benefit from a coordinated effort to further develop or refine a multidimensional anhedonia scale and/or (challenge-based) behavioural tests that are explicitly mapped onto domain-specific processes. It is also worthwhile exploring whether ecological momentary assessment of digital phenotyping could support more precise capturing of anhedonia subdomains in ecologically valid ways.^21^ Importantly, new instruments should be explicitly linked to candidate neurobiological mechanisms, thereby facilitating mechanistically informed trial design.

#### Expanding the neurobiological framework to better understand underlying anhedonia-related phenotypes

Reward symptom research has historically centred on dopaminergic dysfunction.^21^ Although dopamine plays a key role in reward anticipation and motivation, this framework is incomplete. There is a strong need to converge on a biologically informed definition of anhedonia that includes constructs such as reward anticipation/motivation which, based also on preclinical research, are regarded as being more relevant to apathy rather than anhedonia.^22^ Even if anhedonia is often discussed as a widely encompassing term for deficits in both anticipatory and consummatory components of reward processing, these two domains may well rely on (partially) distinct neural substrates. This possible distinction must be recognised in all research. Dopaminergic systems are considered particularly relevant for motivational and effort-related dimensions (often aligned with apathy or anticipatory anhedonia)^23 24^ as well as reward learning,^25–27^ whereas deficits in consummatory anhedonia may additionally involve opioid and endocannabinoid signalling.^4 28^

Attention is also needed for the distinction between tonic and phasic dopamine activity and their differential roles in reward prediction and valuation.^23 24^ Better exploration should separate the role of bottom-up reward dysfunction versus top-down inhibitory mechanisms.^29^ In addition, evidence increasingly implicates noradrenergic, serotonergic, glutamatergic, opioid, inflammatory, immune and metabolic pathways.^3^ Future studies should therefore adopt multimodal approaches integrating functional and structural neuroimaging, electrophysiology and liquid biomarkers.^5^ Neurobiological investigations should move beyond cross-sectional associations and embed biomarkers within longitudinal and interventional designs. This approach may clarify whether distinct anhedonia domains map onto separable neural circuits and molecular systems, and whether early neurobiological changes predict or are accompanied by clinical response.

#### Developmental integration

Development understanding of anhedonia subdomains will benefit from longitudinal study designs which can elucidate the role of illness stage and developmental disease phase. This is essential to disentangle *trait* versus *state* anhedonia aspects, and to examine temporal stability across illness phases and ageing. Age-appropriate and developmentally-appropriate measures and paradigms are necessary to ensure comparability across different (disease) populations involving children, adolescents, adults and elderly individuals. A life-course perspective on anhedonia can help determine to what extent anhedonia functions as a vulnerability marker and/or a consequence of illness progression. Differentiation between minimal and optimal anhedonia data sets would promote standardisation while allowing flexibility for mechanistic depth.

#### Transdiagnostic research

Anhedonia has primarily been considered a symptom of MDD. However, anhedonia is also recognised as a core dimension across multiple psychiatric and neurological conditions, including, for example, schizophrenia, Parkinson’s disease, Alzheimer’s disease, attention deficit hyperactivity disorder (ADHD), traumatic brain injury and bipolar disorder.^10 22^ We need to study anhedonia across different conditions to understand whether it is a single shared biological pathway or a set of similar-looking symptoms driven by different underlying mechanisms. For instance, in Parkinson’s disease disrupted reward processing has been clearly established,^30^ and it is informative to evaluate to what extent prodopaminergic medication in this population influences reward processing, anhedonia and apathy, and whether these effects are independent of motor and cognitive symptom changes. Also, comparisons of treatment effects on anhedonia outcomes across different populations (eg, deep brain stimulation in Parkinson’s disease, obsessive–compulsive disorder and MDD, or prodopaminergic medication in Parkinson’s disease, MDD, schizophrenia) can inform the field on disease-generic versus disease-specific anhedonia mechanisms.

#### Trials explicitly targeting anhedonia

The ultimate goal is that novel insights on the neurobiology underlying anhedonia lead to new interventions that directly target anhedonia. However, novel neurobiological insights may also arise from testing to what extent different existing treatment options lead to a reduction in specific anhedonia aspects. Unfortunately, most existing pharmacological and psychosocial trials in MDD collect information about anhedonia as a secondary outcome with often crude, incomplete assessments of all relevant anhedonia domains and aspects.^16^ New experimental studies are needed that adopt designs explicitly targeting anhedonia as a primary outcome with mechanistically defined treatment targets. For this, innovative designs are needed. A 2×2 factorial approach combining psychosocial (eg, behavioural activation, exercise, exposure-based interventions) and pharmacological strategies could clarify whether modalities preferentially affect specific anhedonia subdomains. Combination treatment approaches of pharmacological plus psychosocial or behavioural activation interventions should test if the non-pharmacological treatment could boost drug effects in anhedonia domains, and using non-pharmacological control groups could help identify specific advantages and risks of pharmacological modulation of hedonic experience. Importantly, treatment strategies should expand beyond monoaminergic paradigms, such as the Fast-fail Trial in Mood and Anxiety Spectrum Disorder (FAST MAS trial) with kappa-opioid receptor antagonist,^31^ and also consider, for example, psychedelics, ketamine and neuromodulation interventions. Pharmacological experiments should consider dose-dependent receptor dynamics and side-effect profiles that may counteract prohedonic effects. Adaptive, basket, umbrella or sequential trial designs could allow efficient testing of mechanism-based interventions. Symptom network analyses may clarify whether improvement in anhedonia mediates broader reductions in depressive or negative symptom burden. In addition to symptom changes, outcomes should include functional recovery, quality of life and real-world engagement.

Trial duration also warrants careful consideration. Anhedonia may respond at different temporal trajectories than overall depressive symptoms. Rapid-acting agents may produce early changes in reward processing, yet functional recovery may require longer periods for relearning reward and motivation contingencies. Trials extending three to 12 months, with predefined adaptive stopping rules, may better capture both early mechanistic shifts and sustained clinical benefit.

### Translational and preclinical research

#### Reverse translation to reach translatable animal–human paradigms

Animal models are useful for causal testing of neural signatures and behavioural readouts identified in humans, and for testing potential novel targeted interventions. Reverse translation should start from patient-level phenotypes and functional outcomes, followed by mapping these onto experimental tasks in healthy volunteers and animals. There is a need to first define the functional consequences of anhedonia that matter to patients, before refining or developing tasks capable of indexing these processes across species. This is fully consistent with the principle that evidence synthesis should clarify what to study next, rather than prematurely declaring efficacy or failure.^32^ For defining effective behavioural tasks, a two-step process should be used. First, a multistakeholder consensus should be developed that reaches a more precise definition of anhedonia based on discrete cognitive and motivational mechanisms. Second, a reverse translation approach should develop novel tasks and models mediated by neurocircuitries that are conserved between humans and other species.

For instance, for reward learning, robust evidence indicates that individual differences in people’s ability to learn from rewards map onto molecular (positron emission tomography), functional (fMRI) and electrophysiological (Event-related potential, ERP) markers of the mesolimbic brain reward pathway, highlighting a promising ‘surrogate’ marker that could be used to stratify or enrich clinical trials.^27^ In line with this, a recent proof-of-concept study that used Probabilistic Rewards Task (PRT) performance (as well as other markers) to prospectively guide antidepressant treatment found that antidepressant response could be boosted by 66.8% (from a response rate of 42.8% in individuals without pretreatment biomarkers to 71.4% in those with biomarkers).^33^ Notably, some of these biological markers (eg, the reward positivity ERP) are conserved across species (eg, rats and humans)^34 35^ and therefore valuable. Also of note, antianhedonic pharmacological challenges or treatments (eg, ketamine) or proanhedonic manipulations (eg, early life adversity, chronic mild stress exposure, reduced dopaminergic signalling via D2/3 autoreceptor activation) induced the same effects in humans and rodents, highlighting strong translation.^27^

Ultimately, translational studies should show transition from showing purely mechanistic insights to providing an early signal of clinical utility. This requires the development of proxy measures of anhedonia that have clear clinical relevance, offer scalability and show reliable and strong correlations with quantitative biological assessments used in preclinical and experimental human research. Such a translational alignment process will provide more translatable animal and human paradigms to measure anhedonia. To advance mechanistic understanding and drug development, assessment tools must enable cross-species translatability and include behavioural and challenge paradigms, reward-learning and reinforcement tasks, and neurobiological readouts (eg, imaging, electrophysiology, biomarkers). Human studies should also incorporate challenge-based paradigms rather than relying solely on passive measures, mirroring preclinical methodologies. Novel protocols could be developed to assess adults in ecologically valid, manualised group settings that elicit and measure social engagement, reward anticipation and motivated participation, analogous to laboratory classroom paradigms in ADHD.^36 37^

Anhedonia can be positioned as a paradigmatic case: rather than asking whether dopaminergic treatments ‘work’, the challenge should be reframed as understanding *which mechanisms, in which patients, measured how*, should be studied next. This shift in thinking is in line with the precision psychiatry focus of the current neuroscience field.^1^

#### Innovation in animal and human models

Human and animal models of anhedonia are being developed, however they lack pharmacological validation as there are no approved treatments for the condition. It is useful to include a positive control intervention. The dopamine agonist pramipexole, which showed efficacy in reducing depression,^38^ could serve as such a positive control when its effects are broadly validated for anhedonia outcomes. So, pramipexole’s effects on tasks such as the PRT may help probe whether existing models are fit for purpose, particularly under chronic rather than acute dosing conditions. But it might be productive to specify the ‘confirmed clinical effects’ of pramipexole, and to further describe the purpose of using the same positive control/intervention in multiple tasks.

Potential biomarkers may show group-level or dose-dependent effects in early clinical studies, but it is important to emphasise that to support real-world clinical utility and eventual regulatory acceptance, these measures must also provide actionable information at the individual patient level, such as stratification or prediction of response. There is first evidence that reward-related markers predict antidepressant responses to dopaminergic treatments.^33 39 40^ Engagement with regulators early in this process is essential, aligning with GALENOS’ emphasis on transparency, bias awareness and coproduction.^41^

#### Cross-species behavioural validation

The importance of quantitative, objective behavioural paradigms that can be meaningfully translated across species is clear. Traditional antidepressant screening assays, such as forced swim or tail suspension tests, are now considered outdated and insufficient to use alone for reward research and drug development. These methods primarily index acute stress reactivity rather than reward processing mechanisms. Preclinical findings derived from the sucrose preference test have similarly poorly translated in humans, including in studies in which the human analogue version of this assay (the Sweet Taste Test) has not differentiated individuals with MDD versus healthy controls.^42^ Instead, effort-based operant tasks assessing willingness to exert physical effort to obtain rewards or reward learning tasks, particularly following conditions of chronic mild stress exposure, were prioritised as current best-practice approaches.^10 43^ A subdomain of anhedonic phenotypes that has received substantial cross-species confluence is reward (reinforcement) learning, which has been typically assessed using the PRT. Critically, harnessing touchscreen technology, functionally identical versions of the PRT have been developed and validated for mice, rats, non-human primates and humans.^27^

Species selection emerged as a critical consideration: while rodent models are well suited for probing subcortical circuitry, non-human primates may ultimately be required to address species-specific receptor distributions and neural circuit architectures relevant to higher-order reward processing. A Wellcome-funded project is currently analysing and making data available on the suitability of various animal models in anxiety, depression and psychosis.^44^

#### Enhancing methodological rigor and reproducibility

Robust experimental design, with adequate (external) validation, transparency in reporting and adherence to pre-registration standards, was identified as essential to reduce bias and enhance confidence in translational inferences. Methodological rigour must be strengthened through appropriate randomisation, blinding, automated testing and data collection, proper power calculation and transparent reporting,^45^ in line with Animal Research: Reporting of In Vivo Experiments (ARRIVE) guidelines for animal studies.^46^ To enhance reproducibility in confirmatory preclinical studies, it is recommended to pre-register study protocols in journals, preprints or online registries such as https://www.animalstudyregistry.org/asr_web/index.action and https://preclinicaltrials.eu. This aligns with international initiatives such as CAMARADES (https://clinical-brain-sciences.ed.ac.uk/camarades), which support rigorous design, transparent reporting and systematic approaches to preclinical evidence synthesis.

### Conclusions: towards a coordinated research agenda for anhedonia

Progress in anhedonia research and translation into precision medicine will depend on coordinated actions from multidisciplinary project teams dedicated to conceptual clarification, measurement harmonisation, translational alignment and innovative trial methodology. Repositioning anhedonia as a mechanistically grounded, transdiagnostic treatment target has the potential to transform therapeutic development and improve functional recovery across neuropsychiatric disorders and beyond. Where possible, outcome measures should not only include self-report but also behavioural tasks to better bridge preclinical and clinical endpoints and assess observable outcomes. Interventional research must move beyond secondary analyses of depression trials and future iterations of the GALENOS LSR on anhedonia should expand across disorders and treatments. To realise such an ambitious plan, close collaboration between academia, industry, regulators, funders as well as representatives from people with lived experience is needed.^47^

## References

[1] Kas MJH, Penninx BWJH, Knudsen GM (2025). Precision psychiatry roadmap: towards a biology-informed framework for mental disorders. Mol Psychiatry.

[2] Treadway MT, Zald DH (2011). Reconsidering anhedonia in depression: lessons from translational neuroscience. Neurosci Biobehav Rev.

[3] Pizzagalli DA (2022). Anhedonia: preclinical, translational, and clinical integration.

[4] Der-Avakian A, Markou A (2012). The neurobiology of anhedonia and other reward-related deficits. Trends Neurosci.

[5] Borsini A, Wallis ASJ, Zunszain P (2020). Characterizing anhedonia: A systematic review of neuroimaging across the subtypes of reward processing deficits in depression. Cogn Affect Behav Neurosci.

[6] Pizzagalli DA (2014). Depression, stress, and anhedonia: toward a synthesis and integrated model. Annu Rev Clin Psychol.

[7] Wong S, Le GH, Phan L (2024). Effects of anhedonia on health-related quality of life and functional outcomes in major depressive disorder: A systematic review and meta-analysis. J Affect Disord.

[8] Rassaby M, Spaulding IG, Stein MB (2026). Examining the relationship between pre-treatment anhedonia and treatment outcomes in individuals with anxiety or depression: A systematic review. J Affect Disord.

[9] Whitton AE, Kumar P, Treadway MT (2023). Distinct profiles of anhedonia and reward processing and their prospective associations with quality of life among individuals with mood disorders. Mol Psychiatry.

[10] Pizzagalli DA (2022). Toward a better understanding of the mechanisms and pathophysiology of anhedonia: Are we ready for translation?. Am J Psychiatry.

[11] Cipriani A, Seedat S, Milligan L (2023). New living evidence resource of human and non-human studies for early intervention and research prioritisation in anxiety, depression and psychosis. BMJ Ment Health.

[12] Smith KA, Downs J, Robinson ESJ (2025). GALENOS approach to triangulating evidence (GATE): transforming the landscape of psychiatric research. Br J Psychiatry.

[13] Tonia T, Higgins JPT, Chiocchia V (2026). The GALENOS approach to triangulating evidence: a structured approach for integrating information from human and animal studies. BMC Med Res Methodol.

[14] Homiar A, Thomas J, Ostinelli EG (2025). Development and evaluation of prompts for a large language model to screen titles and abstracts in a living systematic review. BMJ Ment Health.

[15] Ostinelli EG, Chiocchia V, Macleod M (2023). Pro-dopaminergic pharmacological interventions for anhedonia in depression: protocol for a living systematic review of human and non-human studies. Wellcome Open Res.

[16] Ostinelli EG, Salanti G, Macleod M (2025). Pro-dopaminergic pharmacological interventions for anhedonia in depression: a living systematic review and network meta-analysis of human and animal studies. EBioMedicine.

[17] Zhao S, Ye R, Sen A (2026). On the relationships between apathy, depression and anhedonia. J Neurol Neurosurg Psychiatry.

[18] Hohmann E, Beaufils P, Beiderbeck D (2025). Guidelines for Designing and Conducting Delphi Consensus Studies: An Expert Consensus Delphi Study. Arthroscopy.

[19] Sapperstein MH, Thai M, Ang Y-S (2026). Anhedonia and altered reward responsiveness: Relations to psychosocial functioning. *J Mood Anxiety Disord*.

[20] Treadway MT, Bossaller NA, Shelton RC (2012). Effort-based decision-making in major depressive disorder: a translational model of motivational anhedonia. J Abnorm Psychol.

[21] Smith KA, Blease C, Faurholt-Jepsen M (2023). Digital mental health: challenges and next steps. *BMJ Ment Health*.

[22] Husain M, Roiser JP (2018). Neuroscience of apathy and anhedonia: a transdiagnostic approach. Nat Rev Neurosci.

[23] Zhang C, Dulinskas R, Ineichen C (2024). Chronic stress deficits in reward behaviour co-occur with low nucleus accumbens dopamine activity during reward anticipation specifically. Commun Biol.

[24] Zhang C, Kúkeľová D, Sigrist H (2024). Orphan receptor-GPR52 inverse agonist efficacy in ameliorating chronic stress-related deficits in reward motivation and phasic accumbal dopamine activity in mice. Transl Psychiatry.

[25] Der-Avakian A, D’Souza MS, Pizzagalli DA (2013). Assessment of reward responsiveness in the response bias probabilistic reward task in rats: implications for cross-species translational research. Transl Psychiatry.

[26] Lamontagne SJ, Melendez SI, Olmstead MC (2018). Investigating dopamine and glucocorticoid systems as underlying mechanisms of anhedonia. Psychopharmacology (Berl).

[27] Pizzagalli DA (2026). Probing biomarkers and clinical utility of reward learning across species using the Probabilistic Reward Task: 20 years of findings. Nat Mental Health.

[28] Huys QJM, Cools R, Gölzer M (2011). Disentangling the roles of approach, activation and valence in instrumental and pavlovian responding. PLoS Comput Biol.

[29] Zald DH, Treadway MT (2017). Reward Processing, Neuroeconomics, and Psychopathology. Annu Rev Clin Psychol.

[30] Costello H, Berry AJ, Reeves S (2022). Disrupted reward processing in Parkinson’s disease and its relationship with dopamine state and neuropsychiatric syndromes: a systematic review and meta-analysis. J Neurol Neurosurg Psychiatry.

[31] Krystal AD, Pizzagalli DA, Smoski M (2020). A randomized proof-of-mechanism trial applying the “fast-fail” approach to evaluating κ-opioid antagonism as a treatment for anhedonia. Nat Med.

[32] Wright S, Chiocchia V, Elugbadebo O (2024). The therapeutic potential of exercise in post-traumatic stress disorder and its underlying mechanisms: A living systematic review of human and non-human studies. Wellcome Open Res.

[33] Zhukovsky P, Kuhn M, Borchers LR (2026). A precision medicine trial of bupropion and sertraline for major depressive disorder using a biomarker-guided sequential multiple-assignment design. Nat Ment Health.

[34] Iturra-Mena AM, Kangas BD, Luc OT (2023). Electrophysiological signatures of reward learning in the rodent touchscreen-based Probabilistic Reward Task. Neuropsychopharmacology.

[35] Santesso DL, Dillon DG, Birk JL (2008). Individual differences in reinforcement learning: behavioral, electrophysiological, and neuroimaging correlates. Neuroimage.

[36] Wigal SB, Wigal TL (2006). The laboratory school protocol: its origin, use, and new applications. J Atten Disord.

[37] Wigal SB, Hopkins SC, Koblan KS (2020). Efficacy and Safety of Dasotraline in Children With ADHD: A Laboratory Classroom Study. J Atten Disord.

[38] Browning M, Cowen PJ, Galal U (2025). Pramipexole augmentation for the acute phase of treatment-resistant, unipolar depression: a placebo-controlled, double-blind, randomised trial in the UK. Lancet Psychiatry.

[39] Ang Y-S, Kaiser R, Deckersbach T (2020). Pretreatment Reward Sensitivity and Frontostriatal Resting-State Functional Connectivity Are Associated With Response to Bupropion After Sertraline Nonresponse. Biol Psychiatry.

[40] Whitton AE, Reinen JM, Slifstein M (2020). Baseline reward processing and ventrostriatal dopamine function are associated with pramipexole response in depression. Brain.

[41] Cipriani A, Ioannidis JPA, Rothwell PM (2020). Generating comparative evidence on new drugs and devices after approval. Lancet.

[42] Young JW (2023). Development of cross-species translational paradigms for psychiatric research in the Research Domain Criteria era. Neurosci Biobehav Rev.

[43] Treadway MT, Salamone JD (2022). Vigor, Effort-Related Aspects of Motivation and Anhedonia. Curr Top Behav Neurosci.

[44] Monash University (2025). Landmark Wellcome contract launches new global resource to transform mental health research. https://www.monash.edu/medicine/news/latest/2025-articles/landmark-wellcome-contract-launches-new-global-resource-to-transform-mental-health-research.

[45] Vollert J, Macleod M, Dirnagl U (2022). The EQIPD framework for rigor in the design, conduct, analysis and documentation of animal experiments. Nat Methods.

[46] Percie du Sert N, Hurst V, Ahluwalia A (2020). The ARRIVE guidelines 2.0: Updated guidelines for reporting animal research. PLoS Biol.

[47] Downs J (2025). Beyond the methodological binary: coproduction as the third pillar of mental health science. BMJ Ment Health.

